# Supplementary material for: Selenium Nanoparticles Based on Morinda officinalis Polysaccharides: Characterization, Anti-Cancer Activities, and Immune-Enhancing Activities Evaluation In Vitro
Source: Molecules. 2023 Mar 7;28(6):2426. doi: 10.3390/molecules28062426 (PMC10052065; doi:10.3390/molecules28062426)
Supplement: Supplementary file 1 [file molecules-28-02426-s001.zip › molecules-2245129-supplementary.pdf]

Table S1. RT-PCR primer sequences.

IL-2 F	GTGCTCCTTGTCAACAGCG
IL-2 R	GGGGAGTTTCAGGTTCCTGTA
IL-4 F	ATCATCGGCATTTTGAACGAGG
IL-4 R	TGCAGCTCCATGAGAACACTA
IFN-γ F	ATGAACGCTACACACTGCATC
IFN-γ R	CCATCCTTTTGCCAGTTCCTC
β-actin F	GTGACGTTGACATCCGTAAAGA
β-actin R	GCCGGACTCATCGTACTCC

1

Results

			Size (d.n...	% Intensity:	St Dev (d.n...
Z-Average (d.nm):	66.21	Peak 1:	75.68	100.0	28.08
PdI:	0.117	Peak 2:	0.000	0.0	0.000
Intercept:	0.935	Peak 3:	0.000	0.0	0.000
Result quality	Good				

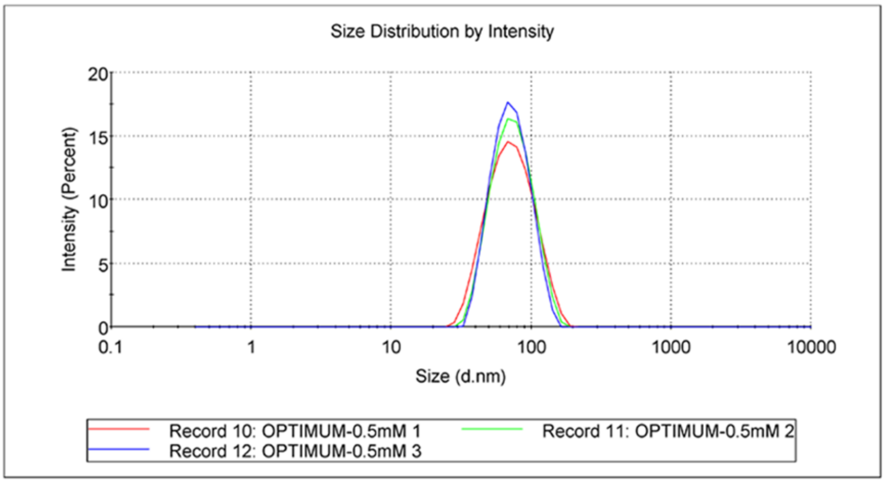

2

Results

			Size (d.n...	% Intensity:	St Dev (d.n...
Z-Average (d.nm):	159.4	Peak 1:	200.8	100.0	74.53
PdI:	0.259	Peak 2:	0.000	0.0	0.000
Intercept:	0.736	Peak 3:	0.000	0.0	0.000
Result quality	Good				

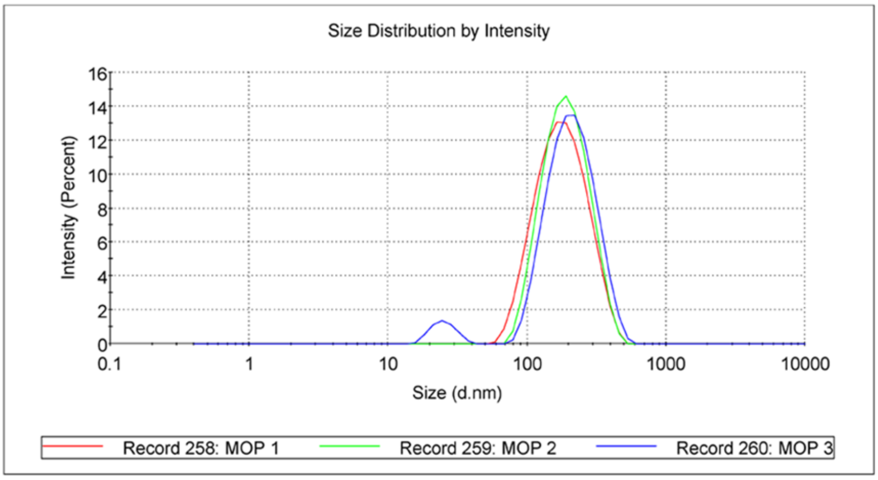

Figure S1. Size distribution report by intensity of Se-MOP (1) and MOP (2).
